# Development of an artificial intelligence-assisted computed tomography diagnosis technology for rib fracture and evaluation of its clinical usefulness

**DOI:** 10.1038/s41598-022-12453-5

**Published:** 2022-05-19

**Authors:** Akifumi Niiya, Kouzou Murakami, Rei Kobayashi, Atsuhito Sekimoto, Miho Saeki, Kosuke Toyofuku, Masako Kato, Hidenori Shinjo, Yoshinori Ito, Mizuki Takei, Chiori Murata, Yoshimitsu Ohgiya

**Affiliations:** 1 Department of Radiology, Showa University, 1-5-8 Hatanodai, Shinagawa-ku, Tokyo, 142-8666 Japan; 2 Fujifilm Corporation, Nishiazabu 2-Chome, Minato-ku, Tokyo, 26-30 Japan

**Keywords:** Diseases, Medical research

## Abstract

Artificial intelligence algorithms utilizing deep learning are helpful tools for diagnostic imaging. A deep learning-based automatic detection algorithm was developed for rib fractures on computed tomography (CT) images of high-energy trauma patients. In this study, the clinical effectiveness of this algorithm was evaluated. A total of 56 cases were retrospectively examined, including 46 rib fractures and 10 control cases from our hospital, between January and June 2019. Two radiologists annotated the fracture lesions (complete or incomplete) for each CT image, which is considered the “ground truth.” Thereafter, the algorithm’s diagnostic results for all cases were compared with the ground truth, and the sensitivity and number of false positive (FP) results per case were assessed. The radiologists identified 199 images with a fracture. The sensitivity of the algorithm was 89.8%, and the number of FPs per case was 2.5. After additional learning, the sensitivity increased to 93.5%, and the number of FPs was 1.9 per case. FP results were found in the trabecular bone with the appearance of fracture, vascular grooves, and artifacts. The sensitivity of the algorithm used in this study was sufficient to aid the rapid detection of rib fractures within the evaluated validation set of CT images.

## Introduction

A rib fracture is commonly encountered in clinical practice. It occurs in 50% of patients who experience blunt chest trauma. In addition to pain, new rib fractures pose a risk of pneumothorax and pulmonary contusion in one-third of patients^1,2^. Multiple rib fractures are often observed in emergency medicine; however, reading computed tomography (CT) images may be outside the expertise of emergency physicians. Diagnostic discrepancies between emergency physicians and radiologists have been reported in 3.2 and 7.2 cases per 1000 CT images of the head and chest, respectively^3^. Radiologists can provide support to emergency physicians in the interpretation of CT images. However, the possibility of missed findings depends on the radiologist’s experience and whether the radiologist-in-charge is a staff or resident radiologist^4–6^.

There have been more diagnostic images in recent years due to the improved performance and multifunctionality of CT, magnetic resonance imaging, and other modalities, leading to the increased workload of reading physicians. Diagnosis and treatment should be promptly provided to patients in the emergency department; inevitably, an adequate image reading cannot be performed in some cases. CT is commonly used in chest trauma since it is helpful for the simultaneous evaluation of lung fields, bones, and soft tissues; sometimes, rib fractures are barely visible^7^. Approximately 20% of rib fractures are not identified on axial section images; therefore, it is important to examine multiplanar reconstructed images, including coronal and sagittal sections, in the search for rib fractures^1^. This process is significantly time-consuming and labor-intensive for both radiologists and other medical specialists because each rib should be examined in all its cross-sections and in three dimensions.

Artificial intelligence (AI), including deep learning, is attracting attention as a medical application in clinical practice. AI technology is undergoing continuous improvements and is expected to reduce the burden of image reading and prevent oversights in trauma patients^8–13^. In this study, the performance of a computer-aided diagnosis (CAD) system was developed and evaluated to detect rib fractures automatically on CT images as the first target for trauma diagnosis support.

## Methods

The design of this retrospective study was reviewed and approved by Showa University Research Ethics Review Board (approval number 2933). The requirement for informed consent was waived by Showa University Research Ethics Review Board owing to the retrospective nature of the study. All methods were performed in accordance with relevant guidelines and regulations.

### Rib fracture CAD

This software (name to be determined, not available for clinical use as a medical device in Apr 2020), developed by Fujifilm Corporation (Tokyo, Japan), had already undergone training using data from another facility^14^.

#### Learning method

**Figure 1:**
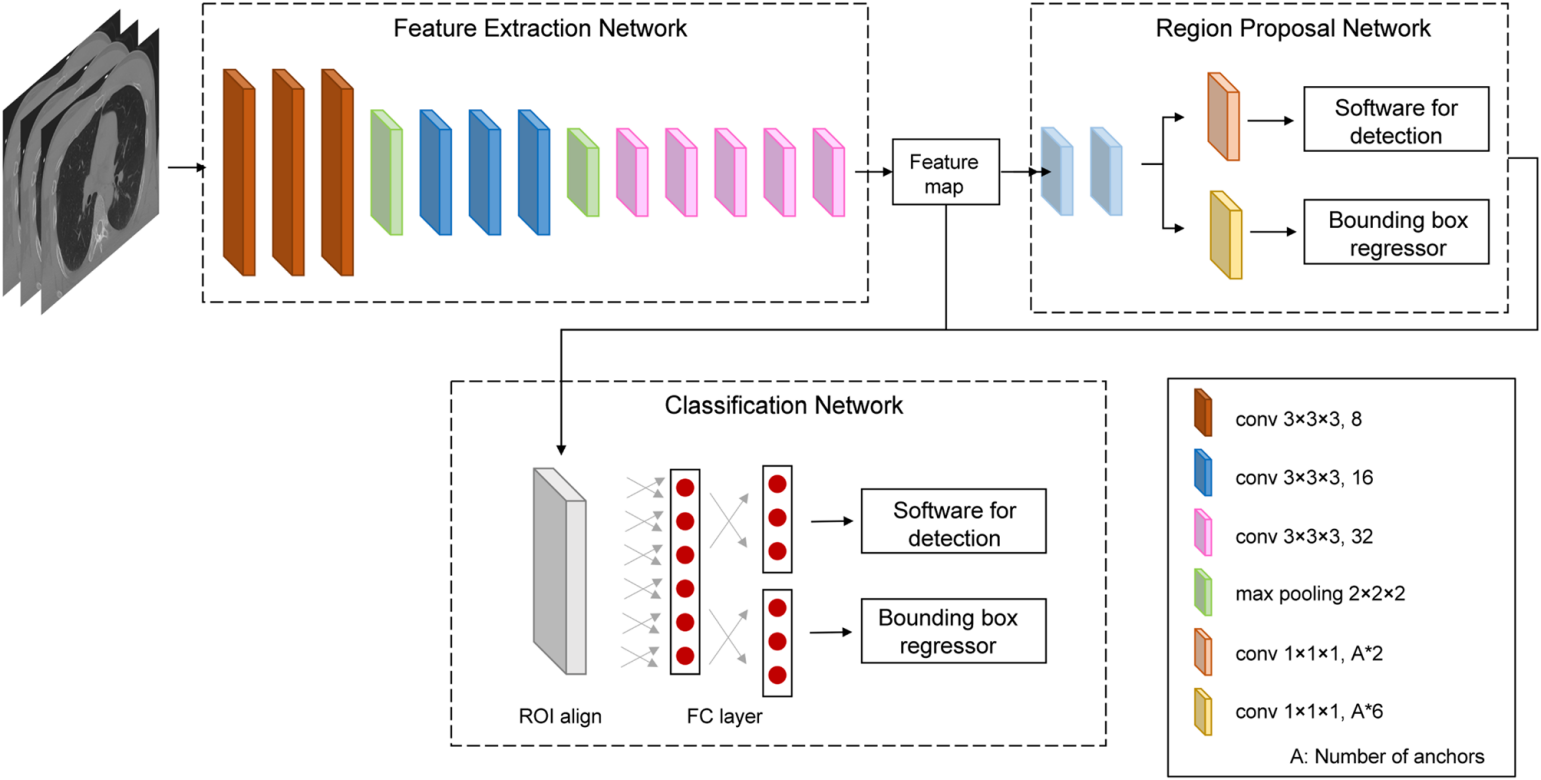
CNN architecture design. From left to right, the legend on the lower right shows the type of each layer (convolution or max pooling), kernel size, and the number of channels.

In this study, a three-dimensional (3-D) object detection network based on a two-stage object detection framework was used (Fig. 1)^14^. A 3-D convolution was applied to the network to maintain 3-D information for continuity between slices. The input image of this network was a chest CT image normalized to x, y, and z = 1.0 mm. The output included the coordinates of the bounding box surrounding the rib fracture and confidence about the presence of the fracture. The evaluation metric for the convolutional neural network during training was the mean average precision calculated using a validation dataset consisting of 21 cases randomly selected from the training dataset (these 21 cases were not used for training), and the convolutional neural network associated with the highest mean average precision was used for evaluation.

#### Initial dataset

The CT image data used for algorithm training consisted of 656 cases collected from Miyazaki University Hospital, Miyazaki, Japan^14^. Radiologists evaluated these cases to determine the fracture regions.

### Evaluation dataset and ground truth

The evaluation dataset consisted of the CT images of patients admitted to Showa University Hospital, Tokyo, Japan, between January 2019 and June 2019, with rib fractures confirmed by the radiologists in the imaging report. Similarly, CT images of patients without fractures were also included in the study as control cases. Eligibility criteria included new rib fractures; open or comminuted fractures and images with confusing artifacts were excluded. The CT scanners used included a 64-slice Multi-Detector row CT scanner (Somatom Sensation 64, Siemens, Munich, Germany), 128-slice Multi-Detector row CT scanner (Somatom Definition AS, Siemens, Munich, Germany), and 192-slice Dual Source CT scanner (SOMATOM Force, Siemens, Munich Germany).

Two radiologists with 9 and 6 years of experience annotated the complete and incomplete fractures and their regions on each CT image at their workstations; these were defined as the “ground truth.” There were 56 total cases, 46 with rib fractures and 10 control cases. There were 199 total regions that the radiologists identified as ground truth: 151 complete fractures and 48 incomplete fractures.

### Evaluation method

As an initial evaluation in this study, each CT image was analyzed using the AI algorithm. The findings from the radiologists’ ground truth and algorithm analysis for all cases were compared and established as true positives, false positives (FPs), and false negatives. These results determined the sensitivity for all fractures, complete and incomplete fractures, and the number of FPs per case.

#### Additional learning

The additional training dataset comprised 333 cases from Showa University Hospital, Tokyo, Japan, from January 2019 to June 2019 and differed from the evaluation dataset. The CT images included “rib fracture” in the reading report, confirmed by the radiologist who initially read the images. All new closed rib fractures within the study period were included in the study. Open or comminuted fractures and images with confusing artifacts were excluded. The radiologist with at least 6 years of experience annotated the complete and incomplete fractures in the retraining cases, and the algorithm was retrained with the new data.

#### Evaluation

The developed algorithm was applied to the evaluation dataset. The evaluation was conducted with the method described previously.

## Results

### Preliminary experiments

First, a performance evaluation was conducted using the initial training dataset (Table 1). As a result, 178 regions were detected (sensitivity: 89.4%), including 138 complete fractures (sensitivity: 91.4%) and 40 incomplete fractures (sensitivity: 83.3%). Furthermore, 2.5 FPs were found per case.

**Table 1:** Results of preliminary experiments.

Cases	Ground truths	Detections	Sensitivity	False positives per case
56	199	178	89.4%	2.5
46 rib fractures 10 control cases	Complete fractures: 151 Incomplete fractures: 48	Complete fractures: 138 Incomplete fractures: 40	Complete fractures: 91.4% Incomplete fractures: 83.3%	

### After additional learning

The algorithm’s detection of complete and incomplete fractures changed by further training. It identified 143 regions with complete fractures, with a 94.7% sensitivity. Incomplete fractures were recognized in 43 regions, with an 89.6% sensitivity; there were 40 regions before re-learning with an 83.3% sensitivity. In total, 186 fractures were correctly identified, with a sensitivity of 93.5%; there were 178 regions before re-learning with a sensitivity of 89.4%.

**Figure 2:**
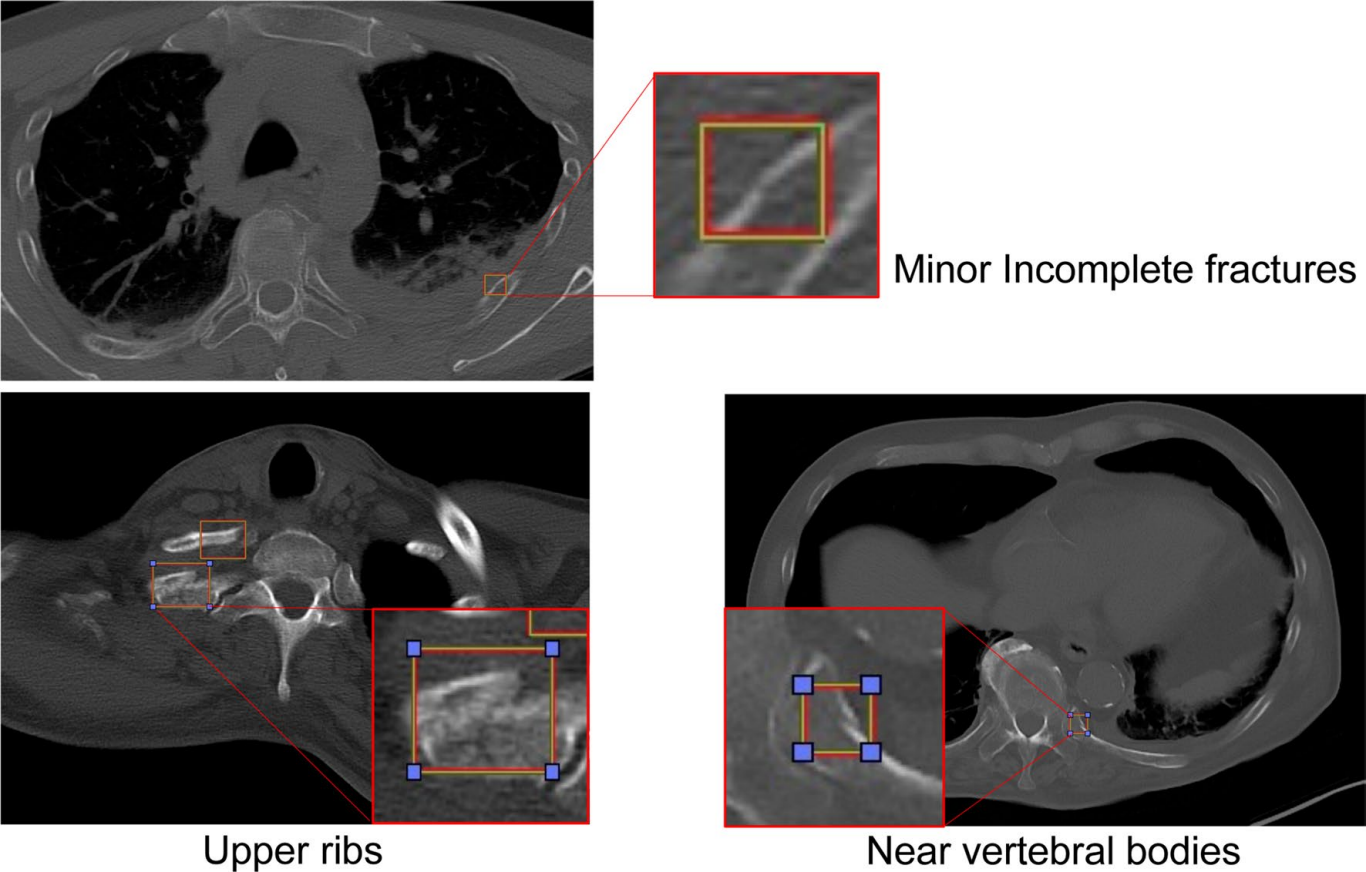
False negative results. These results emerged mainly in the upper ribs, in the proximity of vertebral bodies, and for minor incomplete fractures; additional learning reduced false negatives.

The recognition ability of fractures from the first to the third rib, including the ones involving the lung apex, increased the most with re-learning. Moreover, there was a decrease in the number of false negatives (Fig. 2). The number of FPs per case decreased to 1.9 after relearning compared to the 2.5 FPs before re-learning (Table 2).

**Table 2:** Results after additional learning.

Cases	Ground truths	Detections	Sensitivity	False positives per case
**Re-learning**				
56	199	186	93.5%	1.9
46 rib fractures 10 control cases	Complete fractures: 151 Incomplete fractures: 48	Complete fractures: 143 Incomplete fractures: 43	Complete fractures: 94.7% Incomplete fractures: 89.6%	

## Discussion

**Figure 3:**
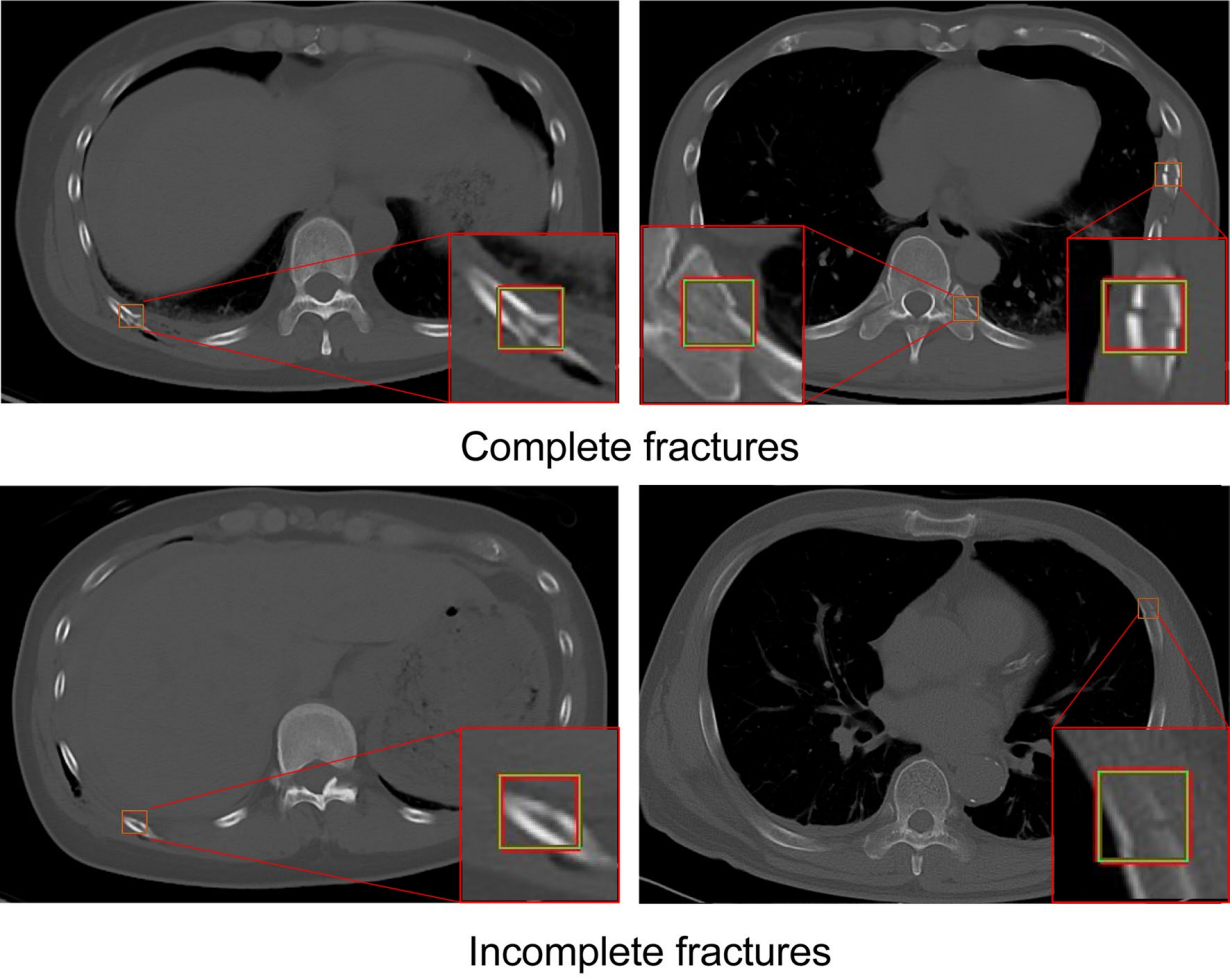
Fractures identified by the algorithm. The algorithm helped identify one case of incomplete fracture, in addition to some complete fractures.

**Figure 4:**
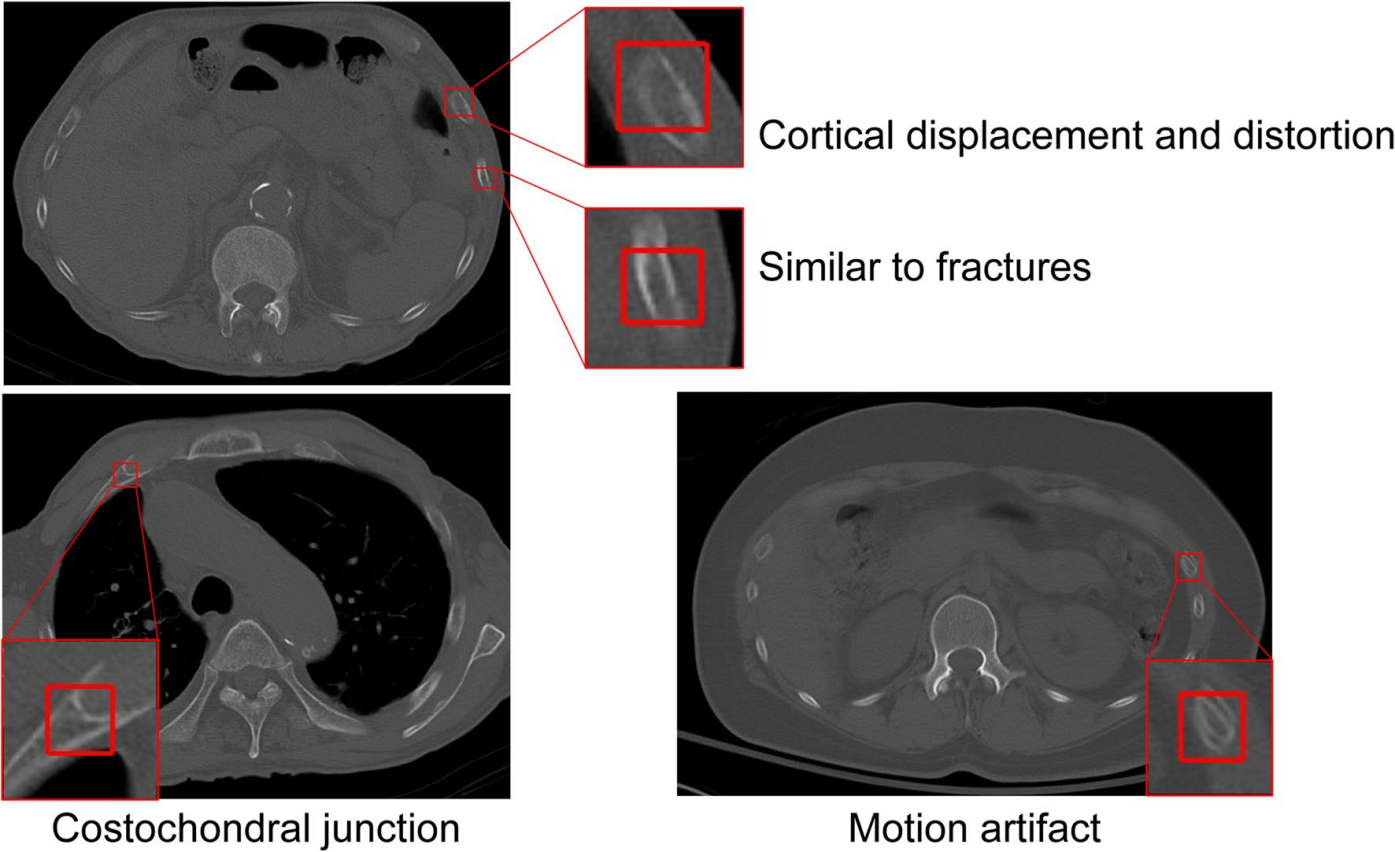
False positive results. These features resembled bone fractures and included strains, vessel grooves, and artifacts.

**Figure 5:**
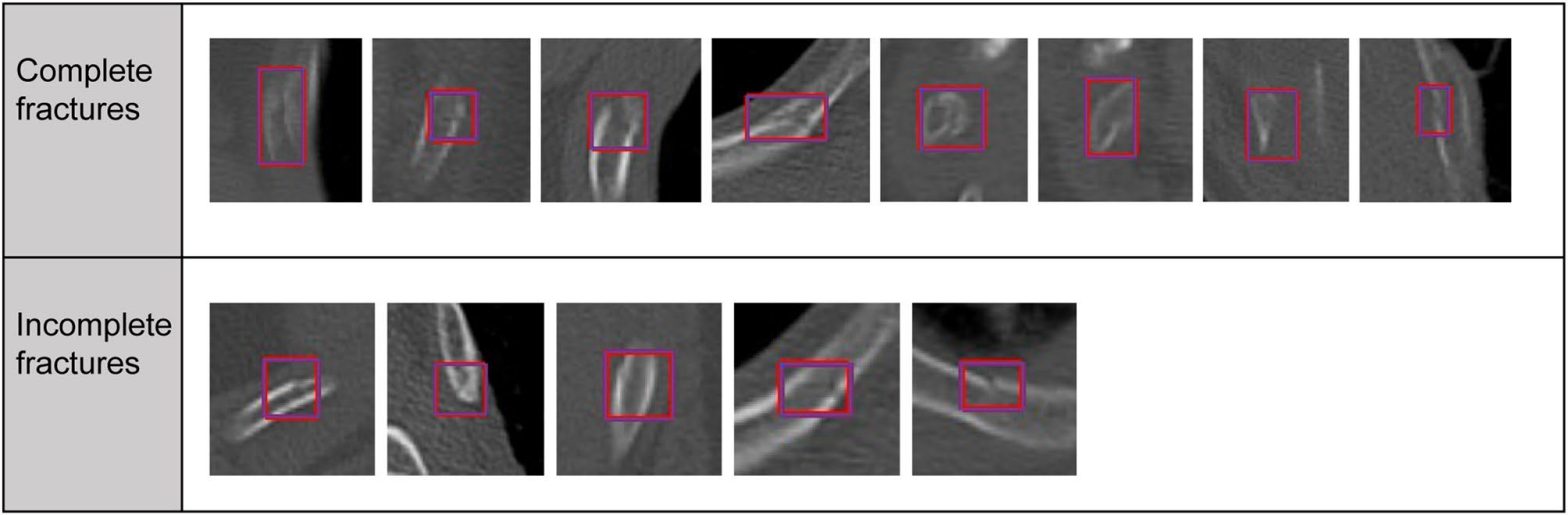
False negative results. These fractures were more frequently unrecognized in the upper ribs and in the proximity of vertebral bodies. It is important to reduce false negative results for clinical application.

Based on the results of the preliminary experiments, the algorithm sensitivity was 89.4%, sufficient for clinical applications (Fig. 3). However, there were some FPs and false negatives. Moreover, the algorithm was less effective in detecting fractures from the first to the third rib (particularly when involving the lung apex), rib fractures near the costovertebral joints, and microfractures (Figs. 4 and 5). Increasing the training data and variation of target findings, such as microfractures near the intervertebral and transverse rib joints and rib fractures, weakly detected before additional training, improved the sensitivity and reduced the number of FPs.

In recent years, the medical applications of AI have been progressing, and their usefulness in the field of emergency medicine and trauma has been widely reported^15,16^. According to Zhou et al.^17^, the average diagnostic sensitivity by radiologists increased to 86.3% with the use of a CAD system (23.9% increase from the radiologist working alone), and the average diagnostic accuracy increased to 91.1% (10.8% increase from the radiologist working alone). Similarly, Zhang et al.^18^ reported that the sensitivity of 82.8–83.9% improved to 88.7–88.9%, and Meng et al.^19^ reported that the accuracy of 81.2–85% improved to 86.3–92.2%. In effect, the use of CAD systems combined with radiologists’ examination resulted in a decrease in FPs and diagnostic time, with an average reduction of 73.9–116 s^17–19^. Furthermore, regarding the AI’s ability to detect rib fractures, Weikert et al.^20^ reported a sensitivity of 65.7% for new and old fractures, and 97 lesions that were not mentioned in the CT reports were identified. Similarly, Jin et al.^6^ reported that AI alone had a sensitivity of 92.9% and an average of 5.27 FPs per scan, compared with a sensitivity of 75.9–79.1% and an average of 0.92–1.34 FPs per scan for radiologists. Hence, the AI and radiologists’ collaboration improved the sensitivity to 94.4% and reduced the time for diagnosis by approximately 86%^6^.

**Figure 6:**
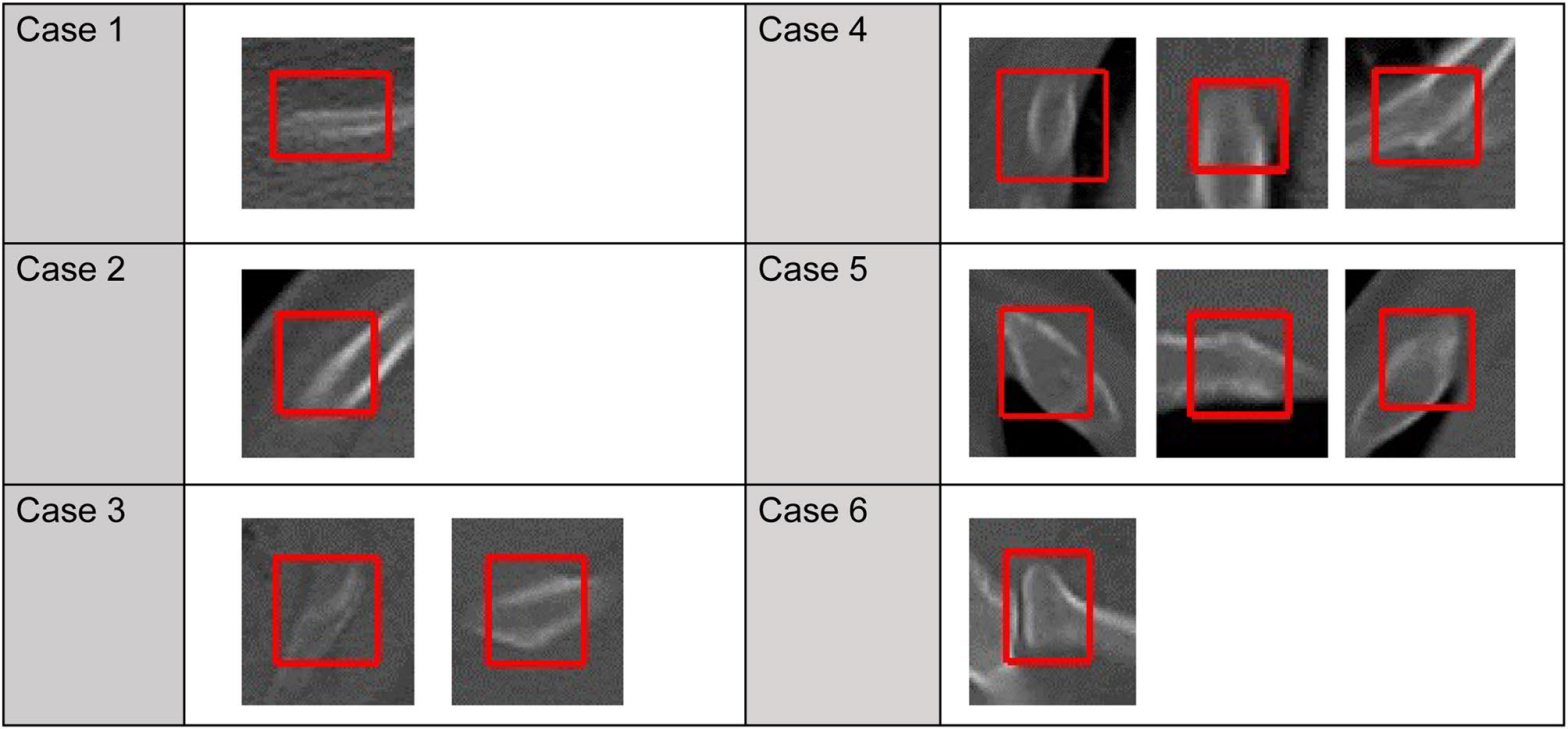
False positive results. These features were classified as fractures in 6 of 10 normal cases.

The newly developed CAD system examined in this study achieved a sensitivity of 93.5%, comparable to that of the systems described in previous reports, using the algorithm alone. However, the CAD system is designed to be a reading aid for the physician rather than a replacement tool^21^ in clinical practice, and further increases in sensitivity are expected. With additional training, the performance of the CAD system improved, with 1.9 FPs per case; this was lower than previously reported values^6^. However, FPs were detected in 6 of the 10 control cases; the features extracted, including deformities of the bone cortex, calcification of the costochondral transition, and osteophytes of the costovertebral joint, may have been due to old fractures (Fig. 6). These FPs could be reduced by training with additional fractures of various shapes and other features that may be erroneously identified as fractures. Interestingly, it has been reported that the FP rate with radiologist-alone diagnosis is lower than that with AI-alone diagnosis. However, the sensitivity of the radiologist-alone diagnosis decreases more than that for the AI-alone diagnosis as the diagnosis time increases^6^. In this study, a CAD system was developed, and it was confirmed that its detection ability is sufficient for clinical practice. The CAD system with the bone number labeling technology developed is expected to reduce the diagnosis time and improve the image interpretation efficiency^22^.

This study had some limitations, starting with its retrospective design. The physician who input the ground truth on the evaluation dataset knew that the CT images were collected to determine rib fractures, even though he did not know the exact location of the rib fractures. This information bias may have made the criteria for rib fracture definition more sensitive than the standard method. The CAD system's sensitivity could be decreasing because of the many ground truths for the radiologists to determine as fractures and the inclusion of ambiguous lesions that are ignored in clinical practice. Moreover, although radiologist annotations are used as correct data, it is sometimes difficult even for experienced radiologists to determine whether a bone discontinuity is a true fracture or a vascular groove. Therefore, there may be FPs and false negatives in the radiologist’s annotation. Furthermore, there may be variabilities due to different facilities. This algorithm's original developer and target facility differed from our institution; hence, the results should not be limited to a single facility. However, the additional training dataset that we used was from the same facility as the evaluation dataset, and differences in results due to the type of CT scanner and different protocols between facilities, including slice thickness, should be considered. The imaging method is standardized in trauma protocols, and the bias due to slice thickness and beam pitch is expected to be inconsequential. Nevertheless, it is necessary to isolate possible differences due to the imaging scanner and protocol and evaluate the results in cases from other facilities and equipment in the future.

In conclusion, the sensitivity of the algorithm used in this study was sufficient to aid the rapid detection of rib fractures within the evaluated validation dataset of CT images. It is important to evaluate the algorithm in a multi-center setting to confirm these findings before using this diagnostic aid in clinical practice.

## Data Availability

The datasets generated and/or analyzed during the current study are available from the corresponding author on reasonable request.
